# Centroid-Based Clustering with *αβ*-Divergences

**DOI:** 10.3390/e21020196

**Published:** 2019-02-19

**Authors:** Auxiliadora Sarmiento, Irene Fondón, Iván Durán-Díaz, Sergio Cruces

**Affiliations:** Departamento de Teoría de la Señal y Comunicaciones, Escuela Técnica Superior de Ingeniería, Universidad de Sevilla, Camino de los descubrimientos, S/N, 41092 Sevilla, Spain

**Keywords:** *αβ*-divergence, *k*-means algorithm, centroid-based clustering, musical genre clustering, unsupervised classification

## Abstract

Centroid-based clustering is a widely used technique within unsupervised learning algorithms in many research fields. The success of any centroid-based clustering relies on the choice of the similarity measure under use. In recent years, most studies focused on including several divergence measures in the traditional hard *k*-means algorithm. In this article, we consider the problem of centroid-based clustering using the family of αβ-divergences, which is governed by two parameters, α and β. We propose a new iterative algorithm, αβ-*k*-means, giving closed-form solutions for the computation of the sided centroids. The algorithm can be fine-tuned by means of this pair of values, yielding a wide range of the most frequently used divergences. Moreover, it is guaranteed to converge to local minima for a wide range of values of the pair (α,β). Our theoretical contribution has been validated by several experiments performed with synthetic and real data and exploring the (α,β) plane. The numerical results obtained confirm the quality of the algorithm and its suitability to be used in several practical applications.

## 1. Introduction

The clustering problem is related to the partition of an analyzed set of samples into a settled number of pairwise disjoint classes or clusters, where samples in the same cluster are more similar to each other than those samples of other clusters. Center-based clustering methods group the samples based on some measure of distance from cluster centers. In this context, the center of a cluster can be a medoid or a centroid. A medoid is the most representative point of a cluster, while a centroid is usually calculated as a minimizer of an optimization problem, with a measure of distortion as the objective function. The choice of a proper measure of similarity or dissimilarity (distance) is a key factor in cluster analysis, since the performance of clustering algorithms greatly relies on this choice.

Arguably, the most popular clustering algorithm is *k*-means with Lloyd’s heuristic, in which squared Euclidean distance is used to compute the distortion. However, there has been a recent burst of interest in extending classical *k*-means algorithm to a larger family of distortion measures. In particular, the use of divergence-based distance functions as a similarity measure has recently gained attention. Research on this topic makes use mainly of two families of divergences, Csiszár *f*-divergences and Bregman divergences. Both families include some well-known divergences. For example, α-divergences, which includes the Kullback-Leibler (KL) divergence, is a type of Bregman and Csiszár *f*-divergences. In fact, α-divergence is the unique class of divergence sitting at the intersection of the Csiszár *f*-divergence and Bregman divergence classes [1]. Other notable Csiszár *f*-divergences are the Hellinger distance and the χ-squared distance [2], whereas the squared Euclidean distance, the Itakura-Saito (IS) divergence and the β-divergence are special cases of Bregman divergences [3].

Such distance functions do not always satisfy certain properties, such as triangular inequality and distance symmetry, making them an improper metric. Thus, for the development of a clustering strategy, one must consider two kinds of centroids obtained by performing the minimization process either on the left argument or on the right argument of the distance function, yielding the left-sided and right-sided centroids, respectively. Closed formulas for both sided centroids computation have been proposed in the literature for different divergence families.

In [4], the classical hard *k*-means algorithm is generalized to the large family of Bregman divergences. The resulting Bregman *k*-means algorithm works for any given Bregman divergence. Since Bregman divergences are not necessarily symmetric, it is necessary to distinguish the two aforementioned sided centroids. It has been shown that the left-centroid is the generalized means of the cluster, also called cluster’s *f*-mean, whereas the right centroid is the center of mass of the cluster, independently of the considered Bregman divergence [5]. For the specific case of α-divergences, closed formulas for the computation of the sided centroids were derived in [6] for the right-type, and in [7,8] for the left-type. Symmetrized centroids have also been derived for clustering histograms in the Bag-of-Words modeling paradigm in [9]. Total Bregman divergences (TDB), which are invariant to particular transformations on the natural space, have also been used for estimating the center of a set of vectors in [10] in the context of the shape retrieval problem. Complete formulation of sided centroids in *k*-means algorithm with TDB are reported in [11]. To the best of our knowledge, there is no closed formulation for the computation of centroids for the whole of Csiszár *f*-divergence family. One of the works that relates Csiszár *f*-divergences and clustering can be found in [12], in which a generalized version of *f*-divergences, called (f,l)-divergence is used for clustering in the particular case of KL-divergence. Finally, other classes of distance functions which are not necessarily Bregman or Csiszár *f*-divergences have been employed for clustering. For example, a notable recent study can be found in [13], in which a *k*-means algorithm using the *S*-divergence is developed for feature coding.

We propose a new center-based clustering algorithm, namely αβ-*k*-means algorithm, using αβ-divergence family as a measure of distortion. Our motivations to explore the family of αβ-divergences for centroid-based clustering are the great flexibility to obtain a rich collection on particular divergences by just tuning the parameters (α,β), and the possibility to yield simple closed formulas for the centroids computation that are of interests for processing several types of data.

αβ-divergences, which were introduced in [14] as a new dissimilarity measure for positive data, have been proven useful in several applications, such as for example the separation of convolved speech mixtures [15], to perform the canonical correlation analysis [16] and noise-robust speech recognition [17]. This family of divergences are governed by two parameters α and β, and cover many of the divergences previously used for clustering, such as α-divergence, β-divergence and KL-divergence.

This is not the first attempt to take into account the αβ-divergences in a clustering approach. In [18], a variation of a k-medoid clustering algorithm is presented based on the αβ-divergences. The resulting algorithm fixes the value of the α parameter to α=1, and varies the value of the parameter β in each iteration through the prominence of the cluster. However, the method is completely different to our proposal. The algorithm we propose in this article computes the centroid of the cluster by solving a minimization problem, whereas the algorithm in [18] obtains the center of the cluster by an exhaustive search optimization technique on the set of the current members of the cluster. Thus, we propose a k-means-type algorithm whereas in [18] it is presented as a k-medoid-type algorithm.

Finally, some authors have pointed out that k-means clustering can be formulated as a constrained matrix factorization problem [19,20]. For instance, in [19] the authors showed that orthogonal symmetric Non-negative Matrix Factorization (NMF) with the sum of squared error cost function is equivalent to Kernel *k*-means clustering. Other variants of NMF, such as Semi-NMF, Convex-NMF, Cluster-NMF and Kernel-NMF are all soft versions of *k*-means clustering [21]. There is also a relationship between NMF based on certain divergences and some clustering approaches. NMF with generalized KL-divergence or *I*-divergence is equivalent to Probabilistic Latent Semantic Indexing (PLSI) [22]. In addition, in [23] it is established that orthogonal NMF based on Bregman divergence problem is equivalent to Bregman hard clustering derived in [4].

This paper is organized as follows: we begin with the formal definition and some properties of αβ-divergences in Section 2. In Section 3 we derive the closed-form formula for the sided centroids by using the αβ-divergence, and generalize the *k*-means algorithm to the αβ-*k*-means algorithm. In Section 4 we demonstrate that the obtained formula for centroid computation match with previous formula for some specific distances and divergences that belong to αβ-divergence family. Section 5 presents some experimental clustering results for synthetic and real datasets. Finally, Section 6 summarizes our main conclusions and provides some suggestions for future research.

## 2. αβ-Divergences

In this section, we recall the definition and some useful properties of the αβ-divergences [14].

**Definition** **1.** *Given two non-negative data matrices of same dimension $P\in R_{+}^{I\times T}$ and $Q\in R_{+}^{I\times T}$, with entries $p_{it}={\left[P\right]}_{it}$ and $q_{it}={\left[Q\right]}_{it}$, the αβ-divergence is given by*

(1) $$D_{AB}^{\alpha ,\beta }\left(P∥Q\right)=\sum_{it}d_{AB}^{\alpha ,\beta }\left(p_{it},q_{it}\right)$$

*where*

(2) $$\begin{array}{c} d_{AB}^{\alpha ,\beta }\left(p_{it},q_{it}\right)=\left\{\begin{array}{ccc} -\frac{1}{\alpha \beta }\left(p_{it}^{\alpha }q_{it}^{\beta }-\frac{\alpha }{\alpha +\beta }p_{it}^{\alpha +\beta }-\frac{\beta }{\alpha +\beta }q_{it}^{\alpha +\beta }\right), & for & \alpha ,\beta ,\alpha +\beta \neq 0\\ \frac{1}{\alpha^{2}}\left(p_{it}^{\alpha }\ln\frac{p_{it}^{\alpha }}{q_{it}^{\alpha }}-p_{it}^{\alpha }+q_{it}^{\alpha }\right), & for & \alpha \neq 0,\beta =0\\ \frac{1}{\alpha^{2}}\left(\ln\frac{q_{it}^{\alpha }}{p_{it}^{\alpha }}+{\left(\frac{q_{it}^{\alpha }}{p_{it}^{\alpha }}\right)}^{-1}-1\right), & for & \alpha =-\beta \neq 0\\ \frac{1}{\beta^{2}}\left(q_{it}^{\beta }\ln\frac{q_{it}^{\beta }}{p_{it}^{\beta }}-q_{it}^{\beta }+p_{it}^{\beta }\right), & for & \alpha =0,\beta \neq 0\\ \frac{1}{2}{\left(\ln p_{it}-\ln q_{it}\right)}^{2}, & for & \alpha ,\beta =0 \end{array}\right. \end{array}$$

It must be pointed out that some specific choices of (α,β) parameters simplify the αβ-divergence into some known divergences or families of divergences, allowing smooth interpolation between many known divergences. In particular, when α=β=0 the αβ-divergence takes the form of a Log-Euclidean distance

(3) $$D_{AB}^{\left(0,0\right)}\left(P∥Q\right)=D_{E}\left(\log P∥\log Q\right)$$

where $D_{E}$ represents the squared Euclidean distance. When α+β=0, with α,β≠0, the αβ-divergence can also be expressed in terms of a generalized Itakura-Saito distance $D_{IS}$ with an α-zoom of the arguments

(4) $$\begin{array}{ccc} D_{AB}^{\left(\alpha ,-\alpha \right)}\left(P∥Q\right) & = & \frac{1}{\alpha^{2}}D_{IS}\left(P^{.\left[\alpha \right]}∥Q^{.\left[\alpha \right]}\right)\\ & = & \frac{1}{\alpha^{2}}\sum_{it}\left(\log\frac{q_{it}^{\alpha }}{p_{it}^{\alpha }}+\frac{p_{it}^{\alpha }}{q_{it}^{\alpha }}-1\right) \end{array}$$

where $P^{.\left[\alpha \right]}$ denotes the one-to-one transformation that raises each element of the vector P to the power α. When α+β=1 the αβ-divergence reduces to the α-divergence,

(5) $$D_{AB}^{\left(\alpha ,1-\alpha \right)}\left(P∥Q\right)=D_{A}^{\alpha }\left(P∥Q\right)$$

whereas when α=1, it reduces to the β-divergence

(6) $$D_{AB}^{\left(1,\beta \right)}\left(P∥Q\right)=D_{B}^{\beta }\left(P∥Q\right).$$

For α=β=0.5, it is proportional to the Hellinger distance

(7) $$D_{AB}^{\left(0.5,0.5\right)}\left(P∥Q\right)=4D_{H}\left(P∥Q\right).$$

Also, the AB-divergence reduces to the standard KL-divergence for α=1 and β=0,

(8) $$D_{AB}^{\left(1,0\right)}\left(P∥Q\right)=D_{KL}\left(P∥Q\right).$$

Although αβ-divergences are not true metrics, they satisfy some interesting properties (see [14] for details and proofs), such us duality, inversion, and scaling:

1. $D_{AB}^{\alpha ,\beta }\left(P∥Q\right)=D_{AB}^{\beta ,\alpha }\left(Q∥P\right)$ (Duality)
2. $D_{AB}^{-\alpha ,-\beta }\left(P∥Q\right)=D_{AB}^{\alpha ,\beta }\left(Q^{.\left[-1\right]}∥P^{.\left[-1\right]}\right)$ (Inversion)
3. $D_{AB}^{w\alpha ,w\beta }\left(P∥Q\right)=\frac{1}{w^{2}}D_{AB}^{\alpha ,\beta }\left(P^{.\left[-w\right]}∥Q^{.\left[-w\right]}\right)$ (Scaling)

αβ-divergence is more flexible, powerful, and robust against errors and noise than other divergence families, such as the α-divergence and β-divergence [14]. The role of the hyperparameters α and β in the robustness property of the αβ-divergence is described in [14]. Formally, it has been shown that if we assume that the right argument of the divergence, Q is a function of a vector of parameters θ, then

(9) $$\frac{\partial D_{AB}^{\alpha ,\beta }\left(P∥Q\right)}{\partial \theta }=-\sum_{it}\frac{\partial q_{it}}{\partial \theta }\underset{weights}{\underset{︸}{q_{it}^{\alpha +\beta -1}}}\underset{\alpha -zoom}{\underset{︸}{\ln_{1-\alpha }\left(\frac{p_{it}}{q_{it}}\right)}}.$$

In this case, the parameter α can be used to control the influence of large or small ratios $\frac{p_{it}}{q_{it}}$ by the deformed logarithm of order 1−α, while the parameter β provides some control on the weighting of the ratios by scaling factors $q_{it}^{\alpha +\beta -1}$.

## 3. *K*-means Clustering with αβ-Divergences

One of the most popular and well-studied data analysis methods for clustering is *k*-means clustering. Let *X* be a random variable that take values in a finite set $X={\left\{x_{i}\right\}}_{i=1}^{n}\subset R^{T}$. *k*-means clustering aims to split X into *k* disjoint partitions ${\left\{c_{i}\right\}}_{i=1}^{k}$, finding a set of centroids $M={\left\{m_{h}\right\}}_{h=1}^{k}\subset R^{T}$, with $\left|M\right|=k$ and k<n. The standard *k*-means formulation finds the partition $C={\left\{c_{i}\right\}}_{i=1}^{k}$ through the minimization of the sum of the squared Euclidean distances between each sample and its cluster centroid. Formally speaking, for a fixed integer *k*, one can define the following squared Euclidean loss function

(10) $$\begin{array}{c} L\left(M\right)=\min_{c_{1},\cdots ,c_{k}}\sum_{h=1}^{k}\sum_{x_{i}\in c_{h}}{∥x_{i}-m_{h}∥}_{2}^{2}. \end{array}$$

The most popular optimization method for this minimization problem is Lloyd’s algorithm [24], which converges to a stable fixed point that corresponds to a local minimum of the loss function. For a given initial partition, Lloyd’s algorithm finds the partition in a two-step iterative process. In the *assignment* step, each data is assigned with the cluster whose centroid is closest. In the *update* step, the centroids are updated as the arithmetic mean of its assigned points. It is well established that the arithmetic mean is the optimal centroid $m_{h,D_{E}}^{*}$ for the Euclidean distance

(11) $$\begin{array}{c} m_{h,D_{E}}^{*}\equiv \frac{1}{|c_{h}|}\sum_{x_{i}\in c_{h}}x_{i}=\underset{m_{h}}{\arg\min}\sum_{x_{i}\in c_{h}}{∥x_{i}-m_{h}∥}_{2}^{2} \end{array}$$

where $|c_{h}|$ refers to the cardinality of th *h*-th cluster.

We propose in this paper to generalize the standard *k*-means clustering to the αβ-divergences. As in k-means standard technique, our objective is to find the set of centroids M that minimizes the AB-divergence of points in the set X to their corresponding centroids. In this context, the centroid of a cluster is defined as the optimizer of the minimum average αβ-divergence. However, since the αβ-divergences are not symmetrical, one must consider two kinds of centroids obtained by performing the minimization process either on the left argument or on the right argument of the divergences. We shall consider the right-sided centroid, $m_{h,D_{AB}}^{R*}$ the optimizer when the minimization process is performed with respect to the right side of the divergence, and the left-sided centroid, $m_{h,D_{AB}}^{L*}$, the optimizer when the minimization is performed with respect to the left

(12) $$\begin{array}{ccc} m_{h,D_{AB}}^{R*}\left(\alpha ,\beta \right) & = & \underset{m_{h}}{\arg\min}\sum_{x_{i}\in c_{h}}D_{AB}^{\alpha ,\beta }\left(x_{i}∥m_{h}\right)=\underset{m_{h}}{arg\min}\sum_{x_{i}\in c_{h}}\sum_{t}d_{AB}^{\alpha ,\beta }\left(x_{it},m_{ht}\right) \end{array}$$

(13) $$\begin{array}{ccc} m_{h,D_{AB}}^{L*}\left(\alpha ,\beta \right) & = & \underset{m_{h}}{\arg\min}\sum_{x_{i}\in c_{h}}D_{AB}^{\alpha ,\beta }\left(m_{h}∥x_{i}\right)=\underset{m_{h}}{arg\min}\sum_{x_{i}\in c_{h}}\sum_{t}d_{AB}^{\alpha ,\beta }\left(m_{ht},x_{it}\right). \end{array}$$

In [5] it is proven that sided centroids with respect to Bregman divergences coincide with the center of mass for the right-type, and with the center of mass for the gradient point set that is a *f*-mean, for the left-type. This implies that the formula for the right-type centroid computation does not depend on the Bregman divergence considered, whereas the formula for the computation of the left-type centroid strongly depends on it. Moreover, the sided centroids for Bregman divergences exhibit different characteristics, and therefore it is necessary to choose between the left and right centroid depending on the application. Contrary to the Bregman divergences case, we can establish a relationship between the sided centroids obtained with αβ-divergences that unifies the optimization process for the sided centroids in a unique problem.

**Lemma** **1.** Let $m_{h,D_{AB}}^{R*}\left(\alpha ,\beta \right)$ denote the optimal right-sided centroid defined in Equation (12) for a given parametrization pair (α,β). The left-sided centroid for the same parametrization is

(14) $$m_{h,D_{AB}}^{L*}\left(\alpha ,\beta \right)=m_{h,D_{AB}}^{R*}\left(\beta ,\alpha \right).$$

**Proof.**  Using the duality property of αβ-divergences, we observe that

(15) $$\begin{array}{ccc} m_{h,D_{AB}}^{L*}\left(\alpha ,\beta \right) & = & \underset{m_{h}}{\arg\min}\sum_{x_{i}\in c_{h}}D_{AB}^{\alpha ,\beta }\left(m_{h}∥x_{i}\right)\\ & = & \underset{m_{h}}{\arg\min}\sum_{x_{i}\in c_{h}}D_{AB}^{\beta ,\alpha }\left(x_{i}∥m_{h}\right)=m_{h,D_{AB}}^{R*}\left(\beta ,\alpha \right). \end{array}$$

□

This last result allows us to formulate the following theorem.

**Theorem** **1.** (Sided αβ-centroids) The right-sided $m_{h,D_{AB}}^{R*}\left(\alpha ,\beta \right)$ and left-sided $m_{h,D_{AB}}^{L*}\left(\alpha ,\beta \right)$αβ-centroid coordinates of a set of point $x_{i}\in c_{h}$ are:

(16) $$\begin{array}{c} m_{h,D_{AB}}^{R*}\left(\alpha ,\beta \right)=\left\{\begin{array}{ccc} {|c_{h}|}^{-\frac{1}{\alpha }}{\left(\sum_{x_{i}\in c_{h}}x_{i}^{\alpha }\right)}^{\frac{1}{\alpha }} & for & \alpha \neq 0\\ \prod_{x_{i}\in c_{h}}{\left(x_{i}\right)}^{\frac{1}{|c_{h}|}} & for & \alpha =0 \end{array}\right. \end{array}$$

(17) $$\begin{array}{c} m_{h,D_{AB}}^{L*}\left(\alpha ,\beta \right)=\left\{\begin{array}{ccc} {|c_{h}|}^{-\frac{1}{\beta }}{\left(\sum_{x_{i}\in c_{h}}x_{i}^{\beta }\right)}^{\frac{1}{\beta }} & for & \beta \neq 0\\ \prod_{x_{i}\in c_{h}}{\left(x_{i}\right)}^{\frac{1}{|c_{h}|}} & for & \beta =0. \end{array}\right. \end{array}$$

The proof is reported in the Appendix A. Please note that the expression obtained for α=0 in Equation (16) corresponds to the limit when α→0 evaluated with L’Hôpital’s rule, in the same way as with β=0 in Equation (17). Closed-form formulas presented in Theorem 1 are essential, since it allows us to develop efficient *k*-means algorithms using αβ-divergences.

Now, we can introduce the following iterative Algorithm 1, known as the αβ-*k*-means algorithm. As in the traditional *k*-means algorithm, the algorithm begins with an initial guess of the centroids (usually at random), and then alternates between the assignment and update steps. In the assignment step, each data point is assigned to the closest cluster centroid, measuring the distance through the αβ-divergence. In the updated step, the centroids are computed using the results on Theorem 1. The algorithm is reiterated until convergence is met. In practice, we can control the stopping criterion by taking the difference between the cost function of two successive iterations. If it is less than a prescribed threshold ϵ the algorithm will stop. A precise definition of the aforementioned strategy using the right-type centroid is presented in Algorithm 1.

**Algorithm 1**αβ-divergence clustering. **Input:** Set $X={\left\{x_{i}\right\}}_{i=1}^{n}\subset R_{+}^{T}$, hyperparameters (α,β), number of clusters k. **Output:**
$M_{\alpha \beta }^{R*}$, local minimizer of $L_{\alpha \beta }\left(M\right)=\sum_{h=1}^{k}\sum_{x_{i}\in c_{h}}D_{AB}^{\alpha ,\beta }\left(x_{i}∥m_{h}\right)$ where $M={\left\{m_{h}\right\}}_{h=1}^{k}$, hard partitioning $C_{\alpha \beta }^{R*}={\left\{c_{h}\right\}}_{h=1}^{k}$ of X. **Method:** Initialize ${\left\{m_{h}\right\}}_{h=1}^{k}$ with $m_{h}\subset R_{+}^{T}$ **repeat**  *(The Assignment Step)*  Set $c_{h}\leftarrow 0,\;1\leq h\leq k$  **for**
i=1 to *n*
**do**   $c_{h}\leftarrow c_{h}⋃\left\{x_{i}\right\}$   where $h=h^{*}\left(x_{i}\right)=\underset{h\prime }{\arg\min}D_{AB}^{\left(\alpha ,\beta \right)}\left(x_{i}∥m_{h\prime }\right)$  **end for**  *(The Update Step)*  **for**
h=1 to *k*
**do**   $m_{h}\leftarrow \left\{\begin{array}{ccc} {|c_{h}|}^{-\frac{1}{\alpha }}{\left(\sum_{x_{i}\in c_{h}}x_{i}^{\alpha }\right)}^{\frac{1}{\alpha }} & \mathrm{for} & \alpha \neq 0\\ \prod_{x_{i}\in c_{h}}{\left(x_{i}\right)}^{\frac{1}{|c_{h}|}} & \mathrm{for} & \alpha =0 \end{array}\right.$  **end for** **until** convergence return $M_{\alpha \beta }^{R*}\leftarrow {\left\{m_{h}\right\}}_{h=1}^{k},\;C_{\alpha \beta }^{R*}\leftarrow {\left\{c_{h}\right\}}_{h=1}^{k}$

Obviously, this algorithm can be extended to the left-type centroid in a straightforward manner by just changing the order of the arguments of the αβ-divergence in the assignment step, and using the left-centroid formulas in the update step to obtain the set of centroids $M_{\alpha \beta }^{L*}$ and the hard partitioning of data $C_{\alpha \beta }^{L*}$. However, it is easy to check that there is a relationship between the αβ-*k*-means algorithm for the right-type centroid and for the left-type centroid. For the same initialization, due to the property the duality of the αβ-divergences and the result of Lemma 1 one can get that $M_{\alpha \beta }^{L*}=M_{\beta \alpha }^{R*}$ and $C_{\alpha \beta }^{L*}=C_{\beta \alpha }^{R*}$. Therefore, the behavior of the left-type αβ-*k*-means algorithm in the αβ plane is equal to behavior of the right-type reflected in the line α=β.

### Conditions for the Convergence of Algorithm

Looking for the conditions ensuring the existence of an optimal set of centroids that achieves the minimum of the clustering loss function based on the αβ-divergence, it is necessary to take into account that αβ-divergences are not necessarily convex in the second argument. In particular, the conditions required for convexity depends on the value of α and β as follows [14]

(18) $$\begin{array}{c} \left\{\begin{array}{ccc} \frac{p_{it}}{q_{it}}\geq \exp_{1-\alpha }\left(\frac{1}{\beta -1}\right) & \mathrm{for} & \beta <\min\left\{1,1-\alpha \right\}\\ \mathrm{always}\;\mathrm{convex} & \mathrm{for} & \beta \in \left[\min\left\{1,1-\alpha \right\},\max\left\{1,1-\alpha \right\}\right]\\ \frac{p_{it}}{q_{it}}\leq \exp_{1-\alpha }\left(\frac{1}{\beta -1}\right) & \mathrm{for} & \beta <\max\left\{1,1-\alpha \right\} \end{array}\right. \end{array}$$

where $\exp_{1-\alpha }\left(\cdot \right)$ is a 1−α deformed exponential

(19) $$\begin{array}{c} \exp_{1-\alpha }\left(z\right)=\left\{\begin{array}{ccc} \exp(z) & \mathrm{for} & \alpha =0\\ {\left(1+\alpha z\right)}^{\frac{1}{\alpha }} & \mathrm{for} & \alpha \neq 0\;\;\mathrm{and}\;\;1+\alpha z\geq 0\\ 0 & \mathrm{for} & \alpha \neq 0;\;\mathrm{and}\;\;1+\alpha z<0. \end{array}\right. \end{array}$$

**Figure 1:**
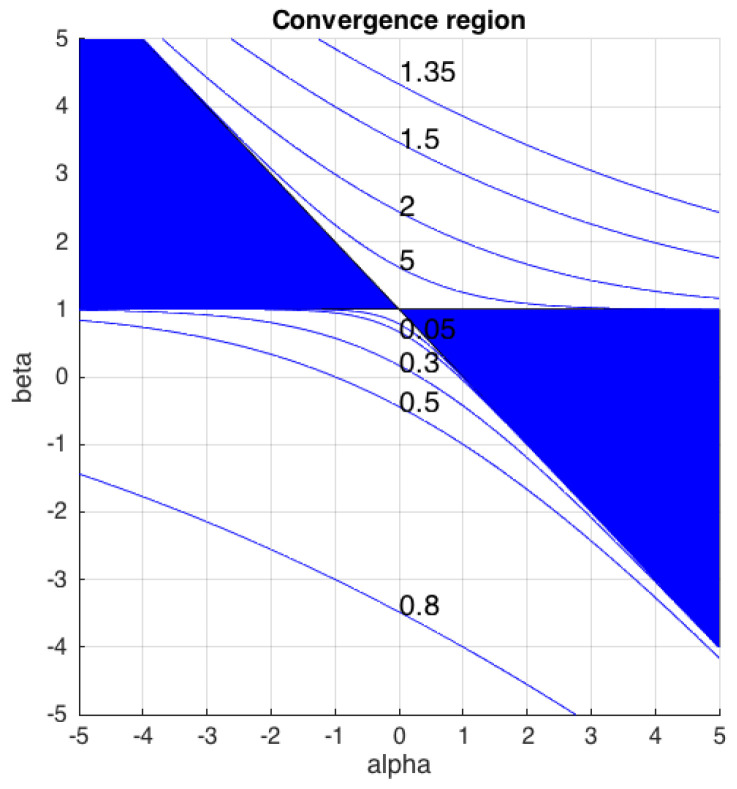
Analysis of the convergence region of the αβ-*k*-means algorithm. The region in blue shows the convex cone that guarantee the convergence of the algorithm to a local minimum for any dataset. Blue lines represent the boundaries of the convergence region for some values of the function $\exp_{1-\alpha }\left(\frac{1}{\beta -1}\right)$.

Figure 1 shows an analysis of the convergence region of the αβ-*k*-means algorithm. The region filled in blue represents the convex cone delimited by the lines α+β=1 and β=1, in which the αβ-divergence is always convex with respect to the second argument. Therefore, the proposed algorithm converges to a local minimum within this cone, independently of the values of the data to be analyzed. However, Equation (18) shows that the convexity of the divergence with respect to $q_{it}$ holds outside the convex cone when the ratios $\frac{p_{it}}{q_{it}}$ are bounded by the function $\exp_{1-\alpha }\left(\frac{1}{\beta -1}\right)$. Therefore, theoretically, the convergence region of the proposed algorithm is greater than the convex cone, and its borders depend on the values that the arguments of the divergence could take. In particular, the maximum and the minimum values of the ratios $\frac{p_{it}}{q_{it}}$ determine the upper and the lower boundaries, respectively. Blue lines in Figure 1 represent the boundaries of the convergence region in the αβ plane for some values of the function $\exp_{1-\alpha }\left(\frac{1}{\beta -1}\right)$. In practice, for relatively small errors between $x_{it}$ and $m_{ht}$, Algorithm 1 is guaranteed to converge to a local minimum in a wide range of (α,β) values.

## 4. Relations with Known Centroid-Based Algorithms

The obtained formulas for sided centroid computation match the previous expressions obtained for various specific divergences and distances. In fact, the above novel algorithm unifies many existing algorithms for *k*-means. For example, apart from the most popular squared Euclidean distance, the algorithm includes those *k*-means algorithms based on α-divergences and β-divergences, as well as the particular cases KL-divergence and IS-divergence.

We shall start with the squared Euclidean distance that can be obtained from the αβ-divergence for α=β=1. By substituting these α and β values on the sided centroids of Equations (16) and (17), we directly obtain that both centroids are the arithmetic mean (11)

(20) $$\begin{array}{c} m_{h,E}^{*}=m_{h,D_{AB}}^{R*}\left(1,1\right)=m_{h,D_{AB}}^{L*}\left(1,1\right)={|c_{h}|}^{-1}\left(\sum_{x_{i}\in c_{h}}x_{i}\right). \end{array}$$

Another interesting case of study is the α-divergence that can be obtained from the αβ-divergences for the parametrization α+β=1. As mentioned before, there are closed formulas for the computation of sided centroids for the *k*-means with α-divergences [7,9]. However, to compare the formulas, it is necessary to take into account that there are two equivalent ways to define the α-divergence family. In particular, some authors employ a slightly different notation that depends on the parameter $\alpha_{A}$ [3], which is related to the parameter α as follows

(21) $$\alpha_{A}=1-2\alpha .$$

The α-divergence parametrized by $\alpha_{A}$ takes the following form

(22) $$\begin{array}{c} D_{A}^{\alpha_{A}}\left(P∥Q\right)=\left\{\begin{array}{ccc} \frac{4}{1-\alpha_{A}^{2}}\sum_{it}\left(\frac{1-\alpha_{A}}{2}p_{it}+\frac{1+\alpha_{A}}{2}q_{it}-p_{it}^{\frac{1-\alpha_{A}}{2}}q_{it}^{\frac{1+\alpha_{A}}{2}}\right), & \mathrm{for} & \alpha_{A}\neq \pm 1\\ \sum_{it}\left(p_{it}\log\frac{p_{it}}{q_{it}}+q_{it}-p_{it}\right), & \mathrm{for} & \alpha_{A}=-1\\ \sum_{it}\left(p_{it}\log\frac{q_{it}}{p_{it}}+p_{it}-q_{it}\right), & \mathrm{for} & \alpha_{A}=1.\; \end{array}\right. \end{array}$$

Closed formulas for sided centroids employing this notation are:

(23) $$\begin{array}{c} m_{h,D_{A}}^{R*}\left(\alpha_{A}\right)=\left\{\begin{array}{ccc} |c_{h}{|}^{-\frac{2}{1-\alpha_{A}}}{\left(\sum_{x_{i}\in c_{h}}x_{i}^{\frac{1-\alpha_{A}}{2}}\right)}^{\frac{2}{1-\alpha_{A}}}, & \mathrm{for} & \alpha_{A}\neq 1\\ \prod_{x_{i}\in c_{h}}{\left(x_{i}\right)}^{\frac{1}{|c_{h}|}}, & \mathrm{for} & \alpha_{A}=1 \end{array}\right. \end{array}$$

(24) $$\begin{array}{c} m_{h,D_{A}}^{L*}\left(\alpha_{A}\right)=\left\{\begin{array}{ccc} |c_{h}{|}^{-\frac{2}{1-\alpha_{A}}}{\left(\sum_{x_{i}\in c_{h}}x_{i}^{\frac{1+\alpha_{A}}{2}}\right)}^{\frac{2}{1+\alpha_{A}}}, & \mathrm{for} & \alpha_{A}\neq -1\\ \prod_{x_{i}\in c_{h}}{\left(x_{i}\right)}^{\frac{1}{|c_{h}|}}, & \mathrm{for} & \alpha_{A}=-1. \end{array}\right. \end{array}$$

Substituting Equation (21) in Equation (4), it is easy to check that $D_{A}^{\alpha_{A}}\left(P∥Q\right)=D_{A}^{\alpha }\left(P∥Q\right)$. Also, substituting Equation (21) in Equations (23) and (24) we obtain

(25) $$\begin{array}{c} m_{h,D_{A}}^{R*}\left(\alpha_{A}\right)=m_{h,D_{AB}}^{R*}\left(\alpha ,1-\alpha \right) \end{array}$$

(26) $$\begin{array}{c} m_{h,D_{A}}^{L*}\left(\alpha_{A}\right)=m_{h,D_{AB}}^{L*}\left(\alpha ,1-\alpha \right). \end{array}$$

One widely employed distance in clustering tasks is the KL-divergence, obtained either for (α,β)=(1,0) from the αβ-divergence, or $\alpha_{A}=-1$ from the α-divergence. In this specific case, the left-sided and right-sided centroids are computed as geometric mean and arithmetic mean respectively [5]. It must be noticed that it is enough to consider (α,β)=(1,0) in Equations (23) and (24) to obtain the same formulas for centroid’s computation than those reported in [5]

(27) $$\begin{array}{c} m_{h,D_{KL}}^{R*}=m_{h,D_{AB}}^{R*}\left(1,0\right)=\frac{1}{|c_{h}|}\sum_{x_{i}\in c_{h}}x_{i} \end{array}$$

(28) $$\begin{array}{c} m_{h,D_{KL}}^{L*}=m_{h,D_{AB}}^{L*}\left(1,0\right)=\prod_{x_{i}\in c_{h}}{\left(x_{i}\right)}^{\frac{1}{|c_{h}|}}. \end{array}$$

Finally, another divergence frequently used in the spectral analysis of speech signals [25] is the IS-divergence. This divergence was first proposed in the context of vector quantization in [26], and can be expressed by the β-divergence, the Bregman divergence and the αβ-divergence for (α,β)=(1,−1). As all the Bregman divergences, the right-sided centroid is computed as the arithmetic mean, but in this case, the left-sided centroid corresponds to the harmonic mean [5]. Again, applying (α,β)=(1,−1) in Equations (23) and (24), we get the same results than in [5]

(29) $$\begin{array}{c} m_{h,D_{IS}}^{R*}=m_{h,D_{AB}}^{R*}\left(1,-1\right)=\frac{1}{|c_{h}|}\sum_{x_{i}\in c_{h}}x_{i} \end{array}$$

(30) $$\begin{array}{c} m_{h,D_{IS}}^{L*}=m_{h,D_{AB}}^{L*}\left(1,-1\right)=\frac{|c_{h}|}{\sum_{x_{i}\in c_{h}}\left(\frac{1}{x_{i}}\right)}. \end{array}$$

## 5. Experimental Results and Discussion

We have evaluated the proposed αβ-*k*-means algorithm on various data types with experiments on both synthetic and real datasets. The first experiment studies the behavior of the algorithm on four different synthetic datasets, whose density is known, whereas the second experiment considers the task of audio genre classification using two different sets of descriptors. The third experiment analyzes the performance of the algorithm in two datasets from the UCI Machine Learning Repository [27]: Iris and Wine. It is expected that the behavior of the algorithm strongly depends on the choice of the tuning parameters and on the type of the data. Given the huge flexibility of the αβ-divergence framework, we must restrict the experiment to some particular cases. Therefore, in our simulations we have varied α and β within the range −2≤α≤2 and −2≤β≤2 with steps of 0.1. In each simulation we run 10 replicates from different randomly selected starting points and determined the partition with the lowest total sum of distances over all replicates. The resulting clusters have been evaluated in terms of accuracy degree (ACC) measured as the number of correctly assigned data points divided by the total number of data. Let us denote $g_{i}$ the ground truth label, $l_{i}$ the clustering assignment produced by the algorithm and $map(l_{i})$ the optimal one-to-one mapping function that permutes clustering labels to match the ground truth labels by using the Hungarian algorithm [28]. The ACC is defined as

(31) $$\begin{array}{c} ACC=\frac{\sum_{i=1}^{n}\delta \left(g_{i},map\left(l_{i}\right)\right)}{n} \end{array}$$

where δ(x,y)=1 if x=y and δ(x,y)=0 otherwise.

### 5.1. Clustering on Synthetic Datasets

**Figure 2:**
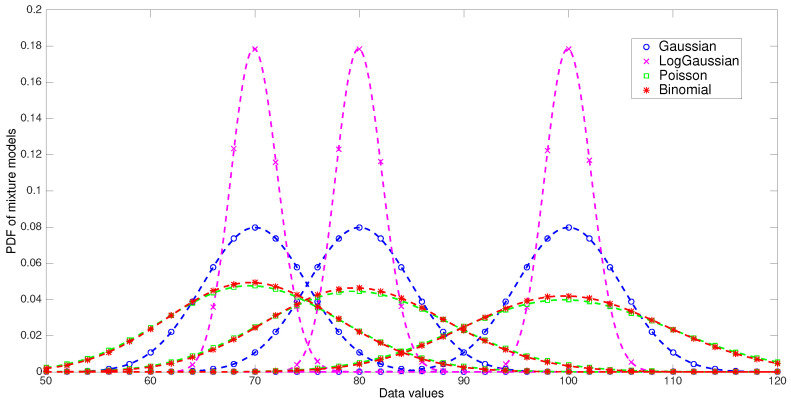
Generative models for dataset used in experiment 1. Each of the four mixture models have three components of Gaussian, Log-Gaussian, Poisson, and Binomial distribution, respectively.

In this experiment we have generated four 1-dimensional datasets of 3000 samples each, based on mixture models of Gaussian, Log-Gaussian, Poisson, and Binomial distributions, respectively. The datasets have three components of 1000 samples each with means equals to 70, 80, and 100, respectively. The standard deviation of the Gaussian and Log-Gaussian densities was set to 5, and the number of trials of the binomial distribution was set to 1000. According this, the variance of the four models are approximately the same. The density functions of the generative models are depicted in Figure 2.

The choice of these density functions is not arbitrary. Gaussian, Poisson, and Binomial distributions are class members of the exponential family of distributions, and in [4] it is well stablished that for every exponential family distribution, it exists a corresponding generalized distance measure. For instance, normal distributions are associated with Euclidean distance, the exponential distribution with IS-divergence and Binomial distribution with KL-divergence. Additionally, Tweedie distributions, which are a particular type of exponential family, are tied to β-divergences [29,30]. Tweedie distributions include Poisson distribution which has been connected to β-divergence with β=0, that is the KL-divergence. Thus, we expect to obtain satisfactory clustering results for some specific divergences in each dataset analyzed.

Furthermore, with the choice of these distributions we want to verify the relation between the convergence region of the algorithm and the extreme values of the different datasets. For example, Poisson and Binomial datasets have similar extreme values and therefore the theoretical convergence region of the algorithm in both cases should be similar. On the contrary, the convergence region for the Log-Gaussian dataset is theoretically more extensive than the convergence regions of the other three datasets considered. In fact, for each cluster it seems probable that the ratio $x_{it}/m_{ht}$ approaches to unit, so that the convergence region covers the whole αβ plane.

**Figure 3:**
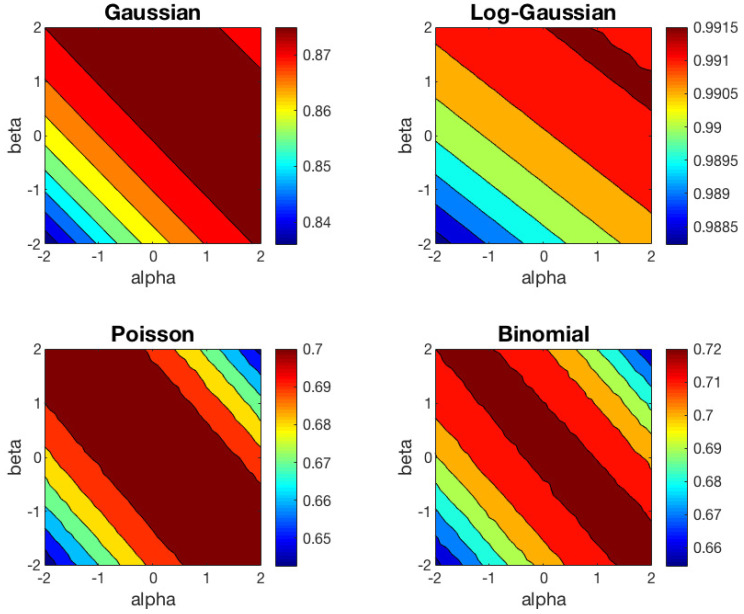
Average ACC obtained with the right centroid αβ-*k*-means algorithm for four different datasets.

**Figure 4:**
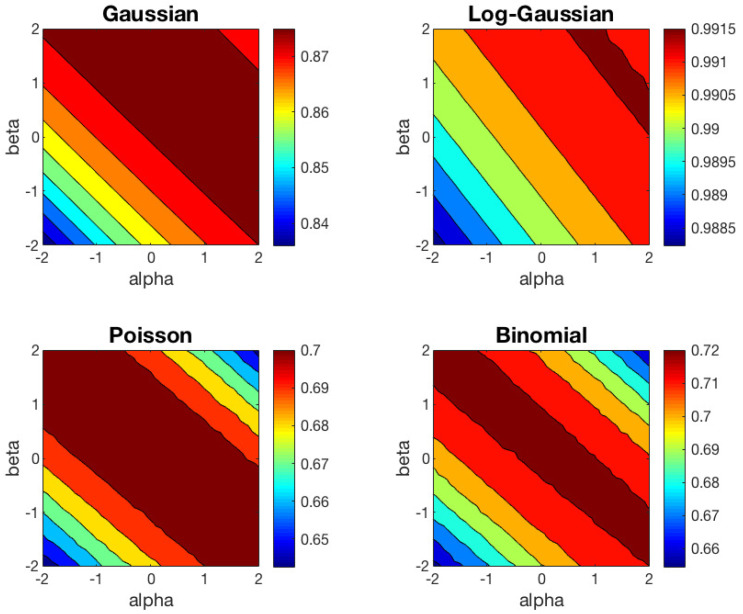
Average ACC obtained with the left-centroid αβ-*k*-means algorithm for four different datasets.

We have repeated the clustering experiment based on 1000 different random datasets, but preserving the same random initialization to run the left and right-type αβ-*k*-means algorithm in each trial. The contour plots of the average ACC results of αβ-*k*-means for the right and left cases are shown in Figure 3 and Figure 4, respectively. In these contour plots, the areas between isolines are filled with constant colors. In Table 1, we show the mean and standard deviation of ACC over 1000 trials obtained for some representative values of the (α,β) pair for the right-type algorithm. We have omitted the values for the left-type since they can be easily inferred from the right-type solutions.

These results can be interpreted from different points of view. First, as we predicted in Section 3, one can check that the behavior of the left-type algorithm is equal to the behavior of the right-type reflected in the line α=β. Second, we observe that the Euclidean distance (α=1,β=1) is well suited to Gaussian dataset, and KL-divergence (α=1,β=0) to Poisson and Binomial datasets, as expected. For the Log-Gaussian dataset, the αβ-*k*-means algorithm behaves practically in the same way in the whole (α,β) region studied. This can be interpreted as the algorithm converges in the whole αβ plane studied, as we had foreseen. Furthermore, for the Gaussian dataset, we observe that the performance of the algorithm begins to decrease significantly for (α,β) values below the line α+β=−2. For the Poisson and Binomial datasets, the region in which the algorithm achieves good results are very similar, although the optimal values of α and β within this region are different for each type of distribution. Finally, we would like to highlight that the regions with good performance for Poisson and Binomial datasets are narrower than the region obtained for the Gaussian dataset.

### 5.2. Musical Genre Clustering

In this experiment we have classified music audio according to the genre of the track. The goal of this experiment is to investigate the optimal divergence for different feature vectors by performing clustering experiments using different (α,β) pairs. Moreover, we have considered two levels of complexity building datasets with K=3 and K=5 genres. The tracks were extracted from the Music Information Retrieval Evaluation eXchange (MIREX) database for US Pop Music Genre Classification that considers a total of 10 genres. Each genre contains 100 tracks of 30-sec duration and the tracks are all 22,050 Hz Mono 16-bit audio files in .wav format. The first built dataset is composed of the genres classical, metal and pop from the MIREX dataset, while the second built dataset adds to the three previous genres country and disco. Therefore, the first dataset is composed of 300 tracks whereas the second dataset is composed of 500 tracks.

Relevant descriptors that could sufficiently discriminate the audio genre were extracted from the audio tracks. Two sets of positive features were analyzed: Discrete-Fourier-Transform-based (DFT-based) descriptors and acoustic spectro-temporal features. The feature extraction process for the computation of the DFT-based descriptors was composed of the following stages [18]. First, the audio segment was divided into *L* overlapping frames of size 2048 points, with 25% overlap between contiguous sections. For each audio track the number of segments obtained was L=430. Then, we performed the DFT of each segment using the Fast-Fourier Transform (FFT) algorithm with Hamming-windowing. After that, we calculated the arithmetic average in each frequency bin considering the absolute values of the complex DFT-vectors, and finally we normalized the average DFT by the sum of the DFT average coefficients. The length of the DFT-based descriptors was 1025.

**Figure 5:**
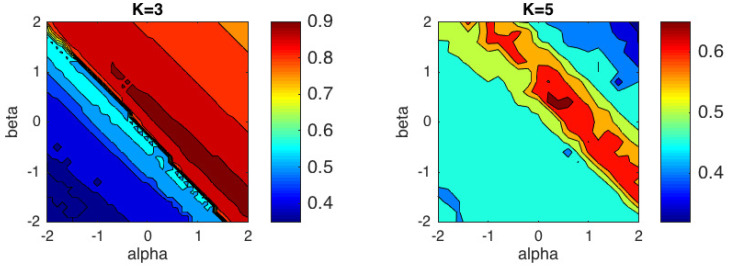
Performance of the αβ-*k*-means algorithm in terms of accuracy for DFT-based descriptors considering K=3 classes and K=5 classes.

Figure 5 shows the performance of the proposed algorithm and Table 2 resumes the results for some specific distances and divergences that belong to the αβ-divergences family. The best performance for K=3, ACC = 0.9767, was obtained for the pair (α,β)=(1.7,−1), which is quite close to the α-divergence. For K=5, the best performance, ACC = 0.6860 was obtained for (α,β)=(0.6,0.4) that is also close to an α-divergence, and in particular to the Hellinger distance.

The acoustics descriptors were composed of temporal features and spectral features. The temporal features extracted were Beat-Per-Minute (BPM) [31], and mean and standard deviation of: Zero-Crossing Rate (ZCR), energy and entropy. The spectral descriptors used were mean and standard deviation of: Spectral Centroid, Spectral Entropy, Spectral Flux, Spectral Flux Centroid, and Spectral Roll-off. The MATLAB Audio Analysis Library [32] was employed for the calculation of all the features except the BPM. The total feature vector consisted on 17 elements.

**Figure 6:**
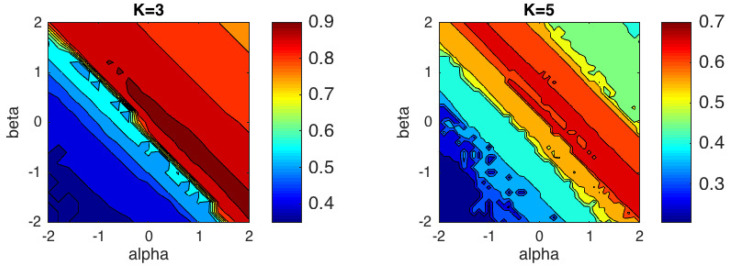
Performance of the αβ-*k*-means algorithm in terms of accuracy for acoustic descriptors considering *K* = 3 classes and *K* = 5 classes.

Figure 6 shows simulation results for the acoustic descriptors for the right-type αβ-*k*-means algorithm. The best performance for K=3, with ACC = 0.9167, was obtained for the pair (α,β)=(0.2,0), which is quite close to the Log-Gaussian distance, while for K=5, the highest value, ACC = 0.7000 was obtained for (α,β)=(0.8,−0.7), which is quite close to IS-divergence. In general, the graph reveals that the region with best performance for this feature vector is close to the generalized IS-divergence, obtained from the αβ-divergence for the values α=−β. Table 3 summarizes the results for some specific divergences and distances obtained with αβ-divergences.

It can be clearly seen from Figure 5 and Figure 6 that the region where the performance is satisfactory is enclosed between the lines α=−β and α+β=1 for the two tested feature vectors. These two lines correspond to the generalized IS-divergence and the α-divergence, respectively. However, a theoretical explanation for this fact is not trivial. As reported in Section 2, the hyperparameters (α,β) can control influence of individual ratios $\frac{x_{it}}{m_{ht}}$ in the centroid computation (see Equation 9). In particular, for α>1 the smaller values of the ratio are down-weighted with respect to the larger ones, whereas for α<1, the larger ratios are down-weighted with respect to the smaller ones. Simultaneously, those ratios are weighted by scaling factors $m_{ht}^{\alpha +\beta -1}$.

In our experiments, one can observe that in the region with best performance the value of α+β seems to be constant and close to unity for the DFT-based descriptors, and to zero for the acoustic descriptors. This cause that the multiplicative weighting factor $m_{ht}^{\alpha +\beta -1}$ does not really affect the estimation of the centroid coordinates for the DFT-based descriptors, whereas the large values in the centroid coordinates are slightly down-weighted compared to the smaller values for the acoustic descriptors. Additionally, we observe that for α+β<0, the performance of the algorithm deteriorates drastically for the two sets of descriptors analyzed, probably due to the inversion of the arguments of the αβ-divergence. It is easy to see that for α+β<0, the inversion property of the αβ-divergence causes small values of the data to be strongly enhanced.

It is worth mentioning here that the values of the hyperparameters that provide the best classification could not be generalized to other classification problems. For other classification tasks, it is preferable to carry out a search on the (α,β) plane to obtain the best performance.

### 5.3. Clustering Analysis in UCI Repository

**Figure 7:**
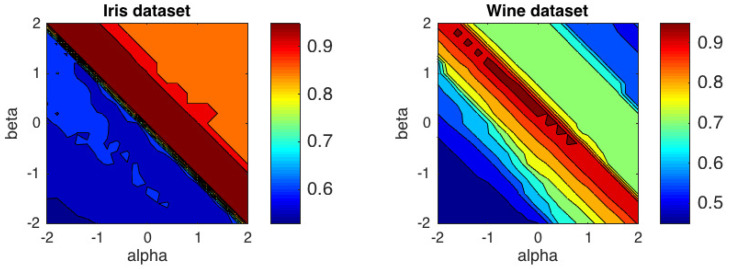
Performance of the αβ-*k*-means algorithm in terms of average accuracy over 50 trials for two UCI datasets: Iris and Wine.

We have conducted experiment over two popular datasets from the UCI repository: Iris and Wine. The Iris dataset consist of three species of iris, 50 specimens in each. The features are measurements in centimeters of the length and the width of the sepals and petals. Wine dataset comprises results of chemical analyses of the content of wine grown in the same region but derived from three different cultivars. The dataset has 13 features and 178 instances. In this experiment we have varied α and β within the range −2≤α≤2 and −2≤β≤2 with steps of 0.2. Figure 7 shows the contour plots of the average accuracy obtained over 50 trials with random initializations. The best performance for Iris dataset, with ACC = 0.9600, was obtained in the (α,β) region delimited by the generalized IS-divergence and the α-divergence. For Wine dataset, the highest value, ACC = 0.9663 was obtained for (α,β)=(−1,1.2), which is quite close to the generalized IS-divergence. In this case, the α-divergence did not get good results. Average ACC levels for some specific distances and divergences are presented in Table 4. It is important to emphasize that the Euclidean distance is not included in the distances and divergences with better results in the two datasets analyzed.

## 6. Conclusions

In this article, we have derived a centroid-based hard clustering algorithm that involves the family of αβ-divergences which are governed by two parameters (α,β) and have many desirable properties useful for clustering tasks. First, αβ-divergences admit closed-form expressions for the computation of the two sided—left or right—centroids, something relevant from the point of view of the implementation of the clustering algorithm. Second, the proposed algorithm, called αβ-*k*-means, unifies many existing implementations of *k*-means obtained with a rich collection of particular divergences that belong to the large family of αβ-divergences. In fact, we have demonstrated that our formulas for sided centroids coincide with other formulas previously developed for some specific cases of the αβ-divergences, such us α-divergences, IS-divergence, and KL-divergence. Finally, the convergence of the algorithm is theoretically assured for a wide region in the (α,β) plane. Although the boundaries of the region of convergence depends on the ratio between the extreme values of the data, in practice, the algorithm seems to work properly outside of that theoretical region.

One of the important and still-open problems is how to tune the parameters (α,β) depending on the distribution of the available dataset and noise or outliers. Our experiments with synthetic datasets have allowed us to verify that the optimal values of the parameters α and β are related to the distribution of the data to be clustered. With this relationship, we can restrict the search range of the α and β values to some αβ-divergences close to other well-known divergences, such us KL-divergence or α-divergences. The derivation of a precise formula for the choice of the parameters α and β is beyond the scope of this work.

Finally, it would be very interesting to study the relationship between the proposed αβ-*k*-means algorithm and the multiplicative NMF algorithm based on the αβ-divergence developed in [14].

## Figures and Tables

**Table 1:** Average and standard deviation of ACC for the synthetic dataset by applying the right-type αβ-*k*-means algorithm for some specific values of (α,β).

(αβ) Parametrization	Generative Model			
	Gaussian	Log-Gaussian	Poisson	Binomial
**(1,1) Euclidean distance**	0.8784 ± 0.0056	0.9915 ± 0.0016	0.6948 ± 0.0143	0.7089 ± 0.0144
**(0,0) Log-Euclidean distance**	0.8754 ± 0.0059	0.9909 ± 0.0017	0.7085 ± 0.0088	0.7216 ± 0.0081
**(1,0) Kullback-Leibler divergence**	0.8783 ± 0.0056	0.9912± 0.0017	0.7057 ± 0.0102	0.7195 ± 0.0092
**(1,−1) Itakura-Saito divergence**	0.8755 ± 0.0059	0.9909± 0.0017	0.7089 ± 0.0088	0.7220 ± 0.0082
**(0.5,0.5) proportional to Hellinger distance**	0.8782 ± 0.0056	0.9913± 0.0017	0.7062 ± 0.0099	0.7199 ± 0.0092

**Table 2:** Clustering results in terms of accuracy of the αβ-*k*-means algorithm for DFT-based descriptors for some specific distances and divergences.

(α,β)	Distance or Divergence	K=3	K=5
(1,1)	**Euclidean distance**	0.5267	0.4700
(0,0)	**Log-Euclidean distance**	0.9233	0.4880
(1,0)	**Kullback-Leibler divergence**	0.9633	0.6200
(1,−1)	**Itakura-Saito divergence**	0.8967	0.4820
(0.5,0.5)	**proportional to the Hellinger distance**	0.9567	0.6560
(1.7,−1)	-	**0.9767**	0.6240
(0.6,0.4)	-	0.9567	**0.6860**

**Table 3:** Clustering results of the αβ-*k*-means algorithm for acoustic descriptors for some specific distances and divergences.

(α,β)	Distance or Divergence	K=3	K=5
(1,1)	**Euclidean distance**	0.8467	0.4980
(0,0)	**Log-Euclidean distance**	0.8833	0.5580
(1,0)	**Kullback-Leibler divergence**	0.8800	0.6420
(1,−1)	**Itakura-Saito divergence**	0.8800	0.5920
(0.5,0.5)	**proportional to the Hellinger distance**	0.8800	0.6460
(0,0.2)	-	**0.9167**	0.6820
(0.8,−0.7)	-	0.9133	**0.7000**

**Table 4:** Clustering results of the αβ-*k*-means algorithm for two UCI datasets: Iris and Wine, for some specific distances and divergences.

(α,β)	Distance or Divergence	Iris Dataset	Wine Dataset
(1,1)	**Euclidean distance**	0.8933	0.7022
(0,0)	**Log-Euclidean distance**	**0.9600**	0.9157
(1,0)	**Kullback-Leibler divergence**	0.9576	0.7135
(1,−1)	**Itakura-Saito divergence**	**0.9600**	0.9157
(0.5,0.5)	**proportional to the Hellinger distance**	0.9536	0.7135
(−1,1.2)	-	**0.9600**	**0.9663**

## Abbreviations

The following abbreviations are used in this manuscript:

ACC	Accuracy
IS	Itakura-Saito
KL	Kullback-Leibler
NMF	Non-negative Matrix Factorization

## Appendix A

In this appendix, we prove the formulated theorem. Without loss of generality, we consider the computation of the right-sided centroid as the minimizer of the following optimization task

(A1) $$\begin{array}{c} m_{h,D_{AB}}^{R*}\left(\alpha ,\beta \right)=\underset{m_{h}}{\arg\min}\left(\sum_{x_{i}\in c_{h}}\sum_{t}d_{AB}^{\alpha ,\beta }\left(x_{it},m_{ht}\right)\right). \end{array}$$

We obtain the solution taking the gradient with respect to the second argument $m_{ht}$, removing all terms independent of $m_{ht}$, and setting it to zero

(A2) $$\begin{array}{ccc} \frac{\partial }{\partial m_{ht}}\left(\sum_{x_{i}\in c_{h}}\sum_{t}d_{AB}^{\alpha ,\beta }\left(x_{it},m_{ht}\right)\right) & = & \sum_{x_{i}\in c_{h}}\frac{\partial \sum_{t}d_{AB}^{\alpha ,\beta }\left(x_{it},m_{ht}\right)}{\partial m_{ht}}\\ & = & \sum_{x_{i}\in c_{h}}\frac{\partial d_{AB}^{\alpha ,\beta }\left(x_{it},m_{ht}\right)}{\partial m_{ht}}=0. \end{array}$$

For the case of α,β,α+β≠0, the partial derivative is given by

(A3) $$\begin{array}{ccc} \sum_{x_{i}\in c_{h}}\frac{\partial d_{AB}^{\alpha ,\beta }\left(x_{it},m_{ht}\right)}{\partial m_{ht}} & = & \sum_{x_{i}\in c_{h}}\frac{\partial }{\partial m_{ht}}\left(-\frac{1}{\alpha \beta }\left(x_{it}^{\alpha }m_{ht}^{\beta }-\frac{\alpha }{\alpha +\beta }x_{it}^{\alpha +\beta }-\frac{\beta }{\alpha +\beta }m_{ht}^{\alpha +\beta }\right)\right)\\ & = & -\frac{1}{\alpha \beta }\sum_{x_{i}\in c_{h}}\left(\beta x_{it}^{\alpha }m_{ht}^{\left(\beta -1\right)}-\beta m_{ht}^{\left(\alpha +\beta -1\right)}\right)\\ & = & \sum_{x_{i}\in c_{h}}m_{ht}^{\left(\beta -1\right)}\left(\frac{m_{ht}^{\alpha }-x_{it}^{\alpha }}{\alpha }\right). \end{array}$$

By setting the derivative in Equation (A3) to zero and considering the non-negative character of $x_{it}$ and $m_{ht}$, we obtain

(A4) $$\begin{array}{ccc} \sum_{x_{i}\in c_{h}}\left(m_{ht}^{\alpha }-x_{it}^{\alpha }\right)=0 & \to & m_{ht}^{\alpha }=\frac{1}{|c_{h}|}\sum_{x_{i}\in c_{h}}x_{it}^{\alpha }\\ & \to & m_{ht}={|c_{h}|}^{-\frac{1}{\alpha }}{\left(\sum_{x_{i}\in c_{h}}x_{it}^{\alpha }\right)}^{\frac{1}{\alpha }},\;\forall t. \end{array}$$

Analogously, for α≠0,β=0, the partial derivative becomes

(A5) $$\begin{array}{ccc} \sum_{x_{i}\in c_{h}}\frac{\partial d_{AB}^{\alpha ,\beta }\left(x_{it},m_{ht}\right)}{\partial m_{ht}} & = & \sum_{x_{i}\in c_{h}}\frac{\partial }{\partial m_{ht}}\left(\frac{1}{\alpha^{2}}\left(x_{it}^{\alpha }\ln\frac{x_{it}^{\alpha }}{m_{ht}^{\alpha }}-x_{it}^{\alpha }+m_{ht}^{\alpha }\right)\right)\\ & = & \frac{1}{\alpha^{2}}\sum_{x_{i}\in c_{h}}\left(-\alpha x_{it}^{\alpha }\frac{m_{ht}^{\left(\alpha -1\right)}}{m_{ht}^{\alpha }}+\alpha m_{ht}^{\left(\alpha -1\right)}\right)\\ & = & \sum_{x_{i}\in c_{h}}m_{ht}^{\left(\alpha -1\right)}\left(\frac{1-x_{it}^{\alpha }m_{ht}^{-\alpha }}{\alpha }\right)=0. \end{array}$$

In this case, the solution is achieved as

(A6) $$\begin{array}{ccc} \sum_{x_{i}\in c_{h}}\left(1-x_{it}^{\alpha }m_{ht}^{-\alpha }\right)=0 & \to & m_{ht}^{\alpha }=\frac{1}{|c_{h}|}\sum_{x_{i}\in c_{h}}x_{it}^{\alpha }\\ & \to & m_{ht}={|c_{h}|}^{-\frac{1}{\alpha }}{\left(\sum_{x_{i}\in c_{h}}x_{it}^{\alpha }\right)}^{\frac{1}{\alpha }},\;\forall t. \end{array}$$

Similarly, for α=−β≠0, the partial derivative takes the following form

(A7) $$\begin{array}{ccc} \sum_{x_{i}\in c_{h}}\frac{\partial d_{AB}^{\alpha ,\beta }\left(x_{it},m_{ht}\right)}{\partial m_{ht}} & = & \sum_{x_{i}\in c_{h}}\frac{\partial }{\partial m_{ht}}\left(\frac{1}{\alpha^{2}}\left(\ln\frac{m_{ht}^{\alpha }}{x_{it}^{\alpha }}+{\left(\frac{m_{ht}^{\alpha }}{x_{it}^{\alpha }}\right)}^{-1}-1\right)\right)\\ & = & \frac{1}{\alpha^{2}}\sum_{x_{i}\in c_{h}}\left(\frac{\alpha m_{ht}^{\left(\alpha -1\right)}}{m_{ht}^{\alpha }}-\alpha x_{it}^{\left(-\alpha \right)}m_{ht}^{\left(-\alpha -1\right)}\right)\\ & = & \sum_{x_{i}\in c_{h}}m_{ht}^{\left(-1\right)}\left(\frac{1-x_{it}^{\alpha }m_{ht}^{-\alpha }}{\alpha }\right)=0 \end{array}$$

which solution takes de same form of Equation (A6).

For α=0,β≠0

(A8) $$\begin{array}{ccc} \sum_{x_{i}\in c_{h}}\frac{\partial d_{AB}^{\alpha ,\beta }\left(x_{it},m_{ht}\right)}{\partial m_{ht}} & = & \sum_{x_{i}\in c_{h}}\frac{\partial }{\partial m_{ht}}\left(\frac{1}{\beta^{2}}\left(m_{ht}^{\beta }\ln\frac{m_{ht}^{\beta }}{x_{it}^{\beta }}-m_{mt}^{\beta }+x_{it}^{\beta }\right)\right)\\ & = & \frac{1}{\beta^{2}}\sum_{x_{i}\in c_{h}}\left(\beta m_{mt}^{\left(\beta -1\right)}\ln\frac{m_{ht}^{\beta }}{x_{it}^{\beta }}+\beta m_{mt}^{\left(\beta -1\right)}-\beta m_{mt}^{\left(\beta -1\right)}\right)\\ & = & \sum_{x_{i}\in c_{h}}m_{ht}^{\left(\beta -1\right)}\left(\frac{\ln m_{ht}^{\beta }-\ln x_{it}^{\beta }}{\beta }\right)=0. \end{array}$$

In this case, the solution is given by

(A9) $$\begin{array}{ccc} \sum_{x_{i}\in c_{h}}\left(\frac{\ln m_{ht}^{\beta }-\ln x_{it}^{\beta }}{\beta }\right) & = & \sum_{x_{i}\in c_{h}}\left(\ln m_{ht}-\ln x_{it}\right)=0\\ & \to & \ln m_{ht}^{|c_{h}|}=\sum_{x_{i}\in c_{h}}\ln x_{it}=\ln\prod_{x_{i}\in c_{h}}x_{it}\\ & \to & m_{ht}=\prod_{x_{i}\in c_{h}}x_{it}^{\frac{1}{|c_{h}|}},\;\forall t. \end{array}$$

Finally, for α=β=0, the partial derivative becomes

(A10) $$\begin{array}{ccc} \sum_{x_{i}\in c_{h}}\frac{\partial d_{AB}^{\alpha ,\beta }\left(x_{it},m_{ht}\right)}{\partial m_{ht}} & = & \sum_{x_{i}\in c_{h}}\left(\frac{1}{2}{\left(\ln x_{it}-\ln m_{ht}\right)}^{2}\right)\\ & = & \frac{1}{2}\sum_{x_{i}\in c_{h}}\left(\frac{2\ln m_{ht}}{m_{ht}}-\frac{2\ln x_{it}}{m_{ht}}\right)\\ & = & \sum_{x_{i}\in c_{h}}m_{ht}^{-1}\left(\ln m_{ht}-\ln x_{it}\right)=0 \end{array}$$

and we get the same solution as in Equation (A9).

After combining the cases in a single expression, the above actualization rules for computing the right-sided centroid become

(A11) $$\begin{array}{c} m_{h,D_{AB}}^{R*}\left(\alpha ,\beta \right)=\left\{\begin{array}{ccc} {|c_{h}|}^{-\frac{1}{\alpha }}{\left(\sum_{x_{i}\in c_{h}}x_{i}^{\alpha }\right)}^{\frac{1}{\alpha }} & \mathrm{for} & \alpha \neq 0\\ \prod_{x_{i}\in c_{h}}{\left(x_{i}\right)}^{\frac{1}{|c_{h}|}} & \mathrm{for} & \alpha =0. \end{array}\right. \end{array}$$

Using Lemma 1, we can directly obtain the formula for the left-sided centroid

(A12) $$\begin{array}{c} m_{h,D_{AB}}^{L*}\left(\alpha ,\beta \right)=\left\{\begin{array}{ccc} {|c_{h}|}^{-\frac{1}{\beta }}{\left(\sum_{x_{i}\in c_{h}}x_{i}^{\beta }\right)}^{\frac{1}{\beta }} & \mathrm{for} & \beta \neq 0\\ \prod_{x_{i}\in c_{h}}{\left(x_{i}\right)}^{\frac{1}{|c_{h}|}} & \mathrm{for} & \beta =0. \end{array}\right. \end{array}$$
